# Hospital Construction

**Published:** 1896-02-29

**Authors:** 


### HOSPITAL CONSTRUCTION.

### PRIVATE HOSPITAL FOR INFECTIOUS DISEASES, NEW YORK.

The proposed new hospital for infectious diseases, for the reception of well-to-do patients who may prefer to make use of such accommodation to the inevitable expenses and worries connected with the nursing of a case of scarlet fever or diphtheria in a private house, will meet a greatly felt want in New York. The initial step towards its establishment is due to the energy of Mrs. John Mintern, of New York, who has herself given 25,000 dols., and has pushed forward the project among her friends with untiring determination.

The plans have been prepared by Messrs. Renwick, Aspinwall, and Renwick, and it is hoped now that the building will progress rapidly. The site chosen is near Avenue C, where the East River curves sharply to the south-west at the foot of East Sixteenth Street, not far from the Willard Parker Hospital. It is bounded on two sides by the river, and will have an area of 260 by 116 feet. The hospital is to consist of three brick buildings separated from each other by an open space of 20 to 25 feet. The central block will be for administration purposes, and two storeys high; here is the accommodation for the matron, the medical staff, and the servants. On the ground floor will be reception and dining rooms, laboratory, dispensary, matron's and doctors' rooms, while the second floor will contain the kitchen, servants' rooms, and bath-rooms.

Two T-shaped buildings complete the hospital on either side the central block. Here the patients' rooms are arranged on each side of a corridor 10 feet wide, two double-bedded rooms and eight single-bedded in each block. The double rooms will measure 17 feet long by 14 wide, the smaller ones 10 by 15 feet. Each will be carefully ventilated and be provided with an open fire-place. At the southern end of both pavilions is a "solarium" for the benefit of convalescents. The number of beds will be 24 in all. The rear end of each of the pavilions is to be, like the central block, two storeys in height, and here will be the rooms for the nurses and attendants.

Between the central block and the pavilions bridges will be built for the conveyance of food from the kitchen to the patients' rooms, 15 feet high, with two tracks for small cars to carry the food. It will be enclosed in metal boxes for sending across, and each box will be disinfected before being returned to the kitchen.

By the charter granted by the Legislature, the hospital is to be under the control of a board of governors, of which the President of the Board of Health and the Health Commissioner will be *ex-officio* members. The staff will consist of two resident physicians, a matron, and eight trained nurses, and a number of attendants. Any patient will be at liberty to be attended by his or her own medical attendant if they so desire.

